# Growth of Ca_x_CoO_2_ Thin Films by A Two-Stage Phase Transformation from CaO–CoO Thin Films Deposited by Rf-Magnetron Reactive Cosputtering

**DOI:** 10.3390/nano9030443

**Published:** 2019-03-15

**Authors:** Biplab Paul, Jun Lu, Per Eklund

**Affiliations:** Thin Film Physics Division, Department of Physics Chemistry and Biology (IFM), Linköping University, SE-58183 Linköping, Sweden; jun.lu@liu.se (J.L.); per.eklund@liu.se (P.E.)

**Keywords:** thin film, nanostructure, Ca_x_CoO_2_, sputtering, phase transformation

## Abstract

The layered cobaltates A_x_CoO_2_ (A: alkali metals and alkaline earth metals) are of interest in the area of energy harvesting and electronic applications, due to their good electronic and thermoelectric properties. However, their future widespread applicability depends on the simplicity and cost of the growth technique. Here, we have investigated the sputtering/annealing technique for the growth of Ca_x_CoO_2_ (x = 0.33) thin films. In this approach, CaO–CoO film is first deposited by rf-magnetron reactive cosputtering from metallic targets of Ca and Co. Second, the as-deposited film is reactively annealed under O_2_ gas flow to form the final phase of Ca_x_CoO_2_. The advantage of the present technique is that, unlike conventional sputtering from oxide targets, the sputtering is done from the metallic targets of Ca and Co; thus, the deposition rate is high. Furthermore, the composition of the film is controllable by controlling the power at the targets.

## 1. Introduction

Thermoelectricity, through its ability to harvest energy from waste heat, can contribute to environmentally friendly energy systems. The thermoelectric efficiency of a material system is determined by a dimensionless parameter, the thermoelectric figure of merit, $ZT=S^{2}/\rho \kappa$, where *S* is the Seebeck coefficient, *ρ* is the electrical resistivity, and *κ* is the thermal conductivity. Therefore, good thermoelectric materials have a high Seebeck coefficient (for high output voltage from a thermoelectric device for a fixed temperature gradient), low electrical resistivity (to reduce energy loss due to Joule heating), and low thermal conductivity (to sustain a high temperature gradient across thermoelectric device for high output voltage) [1,2]. However, the design of such materials is challenging, because electrically conductive materials are generally also thermally conductive.

For the reduction of thermal conductivity without disturbing electronic transport, different nanostructuring approaches have been investigated [3,4,5,6,7,8,9]. In these approaches, different types of nanoscale features have been incorporated. The nanoscale dimension of these features is comparable to the length scale of phonon mean free path, but is higher than electronic mean free path. Therefore, they can selectively scatter phonons without adversely affecting the electronic transport. Thus, *ZT* can be enhanced. Inherently nanolaminated materials [10,11] and artificially layered materials [12,13,14] have been investigated to exploit these concepts. The interfacial phonon scattering in these materials systems is reported to drastically reduce their thermal conductivity, leading to the multifold enhancement of *ZT* [12,13]. The disadvantage of such artificially grown superlattice materials is that they are not thermodynamically stable structures, reducing their stability at high temperature [15]. A related approach is the use of inherently layered materials, e.g., A_x_CoO_2_ (A = Na, Ca, Sr, Ba, La Pr, Nd) [16,17,18,19,20,21,22]. Such layered materials have a layered structure similar to a superlattice structure and can sustain high temperatures.

The layered cobaltates A_x_CoO_2_ consist of alternate stacks of A_x_ sheets and CdI_2_-type CoO_2_ sheets. Due to the inherently layered structure, A_x_CoO_2_ exhibits anisotropic electronic and phononic properties. Among the layered cobaltates, Na_x_CoO_2_ (x~0.7) is reported to have the highest power factor, as high as the standard thermoelectric material Bi_2_Te_3_, due to its low electrical resistivity (*ρ* = 0.2 mΩ·cm at 300 K) and high Seebeck coefficient (100 μV K^−1^ at 300 K) [23]. Even with this high power factor, Na_x_CoO_2_ cannot offer reliable performance, due to its poor chemical stability, because the mobile Na^+^ ions tend to be ejected from the material at high temperatures. To achieve stable performance from this material system, the monovalent Na^+^ ions should be replaced with divalent Ca^2+^ ions, for example by ion exchange method, producing Ca_x_CoO_2_ (0.26 ≤ x ≤ 0.5) thin films [24,25]. The same technique has been reported to be useful to grow a series of layered materials A_x_CoO_2_ (A = Na, Ca, Sr, Ba, La Pr, Nd) [16,17,18,19,20,21,22]. Apart from this ion exchange method, physical [26,27,28,29] methods have also been investigated to grow Ca_x_CoO_2_ thin films.

Here, we have investigated the sputtering/annealing method for the growth of Ca_x_CoO_2_ thin films. In this method, first CaO–CoO thin film is deposited by rf-magnetron reactive cosputtering and then is annealed to form the final phase of Ca_x_CoO_2_. In our previous work, we have demonstrated the growth of Ca_3_Co_4_O_9_ by the sputtering/annealing approach [30,31,32]. The thermally induced phase transformation leading to the final phase of Ca_3_Co_4_O_9_ was found to consist of the following steps:

CoO + O_2_ ⇒ Co_3_O_4_ (1)

Co_3_O_4_ + CaO ⇒ Ca_x_CoO_2_ (2)

Ca_x_CoO_2_ + CaO ⇒ Ca_3_Co_4_O_9_ (3)

Here, we determine how to stop the phase transformation at second step so that final film of Ca_x_CoO_2_ is obtained. Ca_x_CoO_2_ thin films can be promising for near room temperature thermoelectric applications, due to its higher power factor ($S^{2}/\rho$) as compared to Ca_3_Co_4_O_9_ [33].

## 2. Materials and Methods

Three sets of samples with different Ca:Co ratio (0.25, 0.35, 0.45) were prepared. Prior to deposition, Al_2_O_3_ (001) substrates were heated to 650 °C for 1 h under vacuum inside the deposition system, and the same substrate temperature was maintained during deposition. Then, CaO–CoO films were reactively cosputtered from metallic targets of Ca (99.95 % pure) and Co (99.99 % pure) onto the Al_2_O_3_ (001) substrates by rf-magnetron sputtering at 0.27 Pa (2 mTorr) in an oxygen (1.5%)–argon (98.5%) mixture. The deposition system is described elsewhere [34,35]. The power of the cobalt target (50 W) was kept constant, while the power of Ca target was varied to vary the Ca:Co ratio in the films. As-deposited CaO–CoO films were annealed at 650 °C under O_2_ gas flow for 3 h to form the final phase of Ca_x_CoO_2_. The crystal structure and morphology of the films were characterized by θ–2θ XRD analyses using monochromatic Cu Kα radiation (λ = 1.5406 Å), transmission electron microscopy (TEM) by using a FEI Tecnai G2 TF20 UT instrument, from Eindhoven, Netherlands, with a field emission gun operated at 200 kV and with a point resolution of 1.9 Å, and scanning electron microscopy (SEM, LEO 1550 Gemini). The θ–2θ XRD scans were performed with a Philips PW 1820 diffractometer. Ex situ annealing and XRD experiments were performed on a single CaO–CoO thin film with Ca:Co = 0.35. The Ca:Co = 0.35 film was subjected to several sequential annealing steps and θ–2θ XRD scans were performed after each annealing step. The annealing furnace was stabilized at a given set-point temperature prior to inserting the sample for a specified time period. The sample temperature was monitored as a function of time via a thermocouple in contact with the film substrate. The sample was removed from the furnace after the specified annealing time and cooled in ambient air at room temperature. The Ca:Co ratio in the films is confirmed by energy dispersive spectroscopy (EDS) attached to a scanning electron microscope.

## 3. Results and Discussion

### 3.1. As-Deposited CaO–CoO and Annealed Ca–Co–O Films

**Figure 1:**
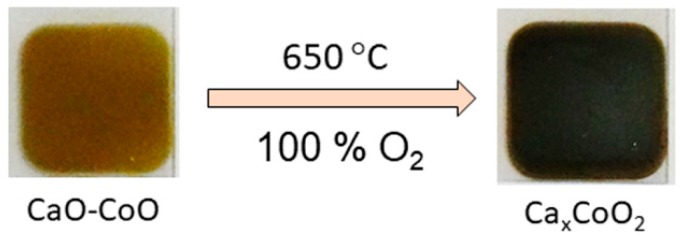
Optical image of as-deposited CaO–CoO film and annealed Ca_x_CoO_2_ film.

**Figure 2:**
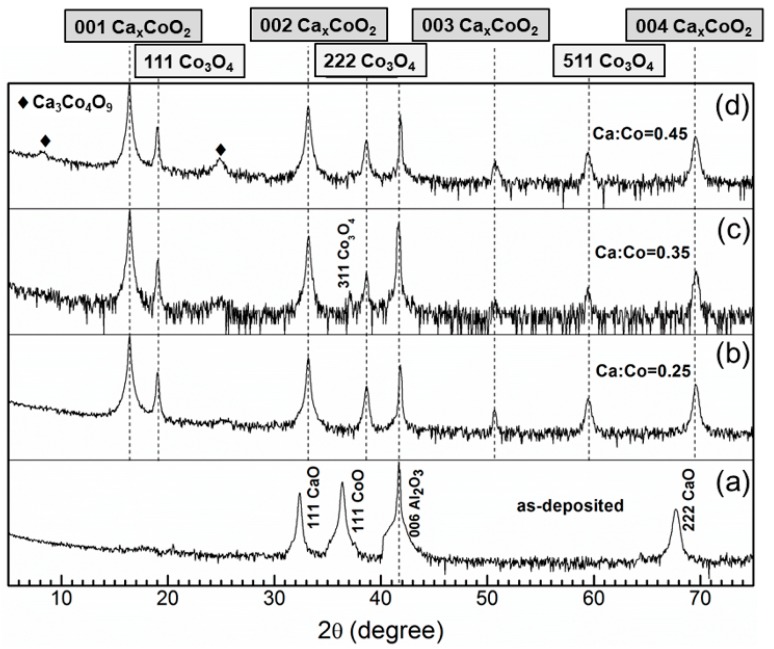
θ–2θ XRD patterns of (**a**) as-deposited CaO–CoO film and annealed films with (**b**) Ca:Co = 0.25, (**c**) Ca:Co = 0.35, (**d**) Ca:Co = 0.45.

The as-deposited films are yellowish in color and turn dark after annealing in oxygen atmosphere (Figure 1). Figure 2a shows a θ–2θ XRD scan for the as-deposited Ca:Co = 0.35 film. XRD scans of all the as-deposited films are similar in appearance (shown in Figure S1 of Supplementary Information), albeit with varying CaO:CoO peak intensity ratios. It is evident from the XRD analyses that as deposited film consists of CaO and CoO phases. Figure 2b–d show the XRD patterns of all the annealed films. These XRD patterns confirm the formation of Ca_x_CoO_2_ phase in all the films and d-spacing is calculated to be 5.434 Å irrespective of the Ca to Co ratio in the films, which nearly matches the value reported elsewhere [24]. Apart from the Ca_x_CoO_2_ phase, the XRD peaks of Co_3_O_4_ are also visible in all the films irrespective of the Ca to Co ratio. The presence of Co_3_O_4_ phase in the film Ca:Co = 0.25 is attributed to the excess Co in the film. The presence of Co_3_O_4_ phase in the post-annealed films Ca:Co = 0.35 and 0.45 is attributed to the inhomogeneous distribution of CaO and CoO, which is also confirmed by TEM analyses of as-deposited films (see below). The small peaks at 2θ angles 8.249 and 26.527° in Figure 2d are attributed to the Ca_3_Co_4_O_9_ phase, which might be due to the local enrichment of the film with the CaO phase.

**Figure 3:**
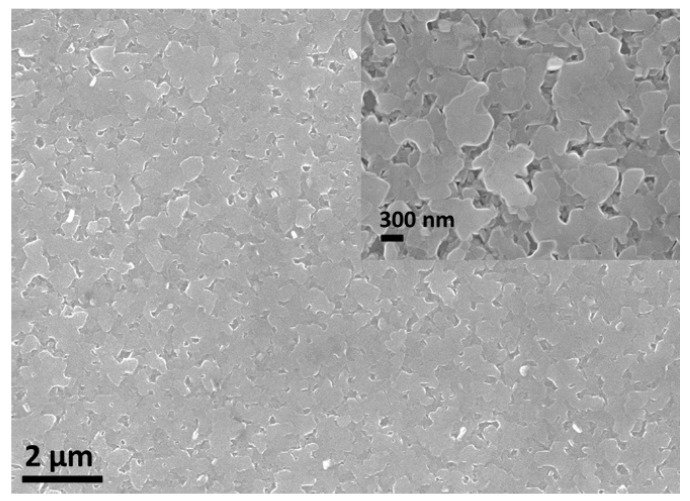
A typical SEM image of the annealed film Ca:Co = 0.35.

Figure 3 shows SEM images of postannealed film Ca:Co = 0.35. The apparent flat surface of the film is attributed to the *c*-axis orientation of the film, which is also consistent with the observation by XRD.

**Figure 4:**
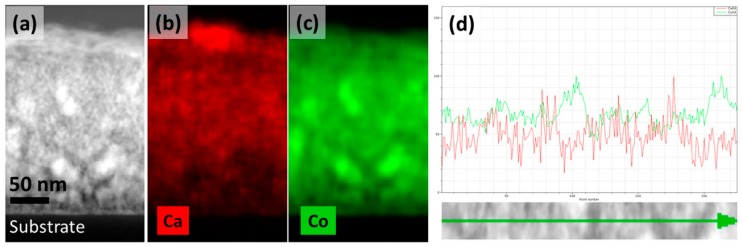
(**a**) TEM image of as-deposited CaO–CoO film, (**b**,**c**) EDS mapping of the corresponding film, (**d**) line scan along in-plane direction of the film; green line represents Co and red line Ca.

Figure 4a shows a typical cross-sectional TEM image of as-deposited film Ca:Co = 0.35. Figure 4b and c show the EDS mapping of the film. The EDS mapping shows the separation into Co-rich and Ca-rich phases, i.e., CoO and CaO. This observation is in consistent with the observation by XRD, i.e., the presence of CaO and CoO phases in as-deposited films. Figure 4b indicates the Ca-deficiency near the interfacial region. This is caused by the segregation of Ca near the surface of the as-deposited films, due to the substrate heating during sputter deposition, consistent with our pervious observation on the growth of Ca_3_Co_4_O_9_ thin films [30]. The inhomogeneous distribution of CaO and CoO phases along the in-plane direction is also confirmed by the line scan as shown in Figure 4d.

**Figure 5:**
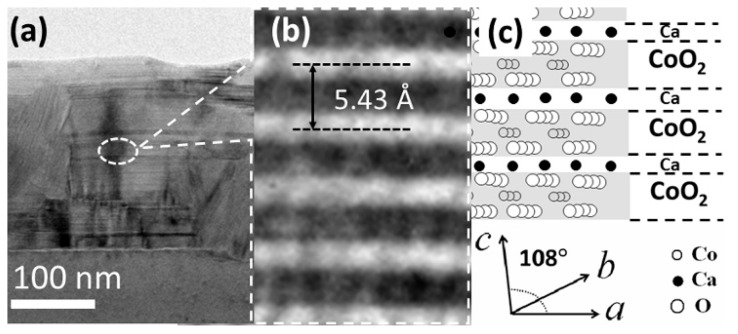
(**a**) TEM image of Ca_0.33_CoO_2_ film, (**b**) Lattice-resolved TEM image and (**c**) schematic of the atomic arrangement of the layers.

Figure 5a shows a TEM image of annealed film Ca:Co = 0.35. The formation of a layered structure is evident from the figure, and there are also grains with nonbasal orientation. The Ca to Co ratio in the layered zone is determined to be ~0.33 by EDS analyses (in TEM), which indicates that the postannealed film Ca:Co = 0.35 is of Ca_0.33_CoO_2_ phase. From EDS analyses, all the postannealed films are found to consist of the same Ca_0.33_CoO_2_ phase irrespective of the Ca:Co ratio in the as-deposited films, which is consistent with the observation by XRD. Figure 5b shows a high resolution TEM (HRTEM) image of the film and d-spacing of the film is confirmed to be 5.43 Å, which is consistent with the value calculated from XRD. Figure 5c schematically shows the atomic arrangements of alternate layers of Ca_0.33_CoO_2._

From the above results, it is concluded that Ca_0.33_CoO_2_ is the favorable composition of Ca_x_CoO_2_ at the present conditions. The phase-purity is likely to improve the Ca_0.33_CoO_2_ film by controlling the composition and ensuring the homogeneous distribution of CaO and CoO phases in the as-deposited CaO–CoO films. It is anticipated that homogeneous distribution of CaO and CoO in as-deposited films can be possible by lowering the deposition temperature.

### 3.2. Ex-Situ XRD Annealing Experiments

**Figure 6:**
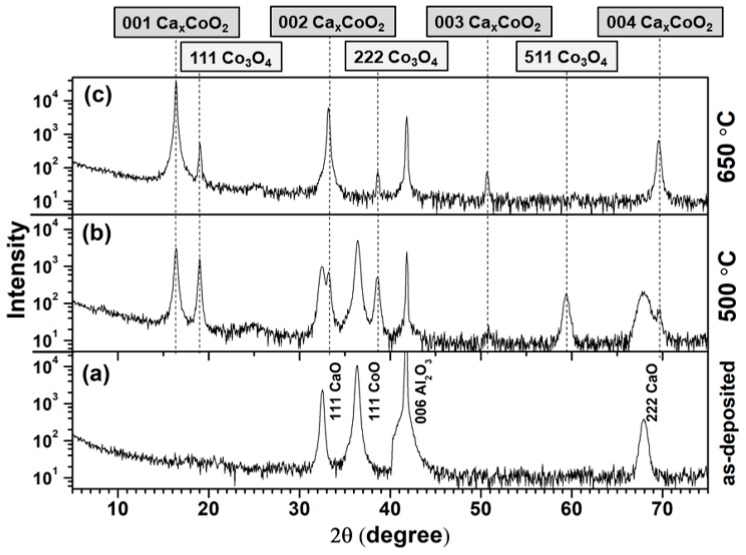
θ–2θ XRD patterns of the Ca:Co = 0.35 thin film as a function of annealing temperature. (**a**) As-deposited film, (**b**) annealed at 500 °C, (**c**) annealed at 650 °C.

Figure 6a shows the θ–2θ XRD scan for the as-deposited film Ca:Co = 0.35. Figure 6b,c show θ–2θ scans after the film was subjected to annealing temperatures 500 °C and 650 °C, respectively. After annealing at 500 °C (Figure 6b), the appearance of Co_3_O_4_ peaks with a concomitant decrease in the peak intensity of CoO indicates the reaction of CoO with oxygen to form Co_3_O_4_. The Co_3_O_4_ has also reacted with CaO to form the Ca_0.33_CoO_2_-phase, as confirmed by the presence of the Ca_x_CoO_2_ 001, 002, 003 and 004 peaks. These results indicate a competition between the formation and consumption of Co_3_O_4_. After annealing at 650 °C (Figure 6c) the Ca_0.33_CoO_2_ phase becomes more intense and CoO phase has disappeared. However, the low-intensity peaks of Co_3_O_4_ still remain. To verify the completion of the phase transformation, the annealing experiment was performed at 650 °C for a longer period (10 h), with no change in XRD peak intensity.

From the above results, it is concluded that two different phase transformation processes (i.e., CoO + O_2_ ⇒ Co_3_O_4_; and Co_3_O_4_ + CaO ⇒ Ca_0.33_CoO_2_) simultaneously occur at temperatures below 650 °C. At 650 °C, the CaO phase is completely consumed by the reaction to form the final phase of Ca_0.33_CoO_2_, and thus the phase transformation processes end. The partial presence of Co_3_O_4_ is attributed to the local enrichment of Co in the film, due to the inhomogeneous distribution of CaO and CoO phases in the film.

## 4. Conclusions

A two-step sputtering/annealing approach has been demonstrated for the growth of Ca_x_CoO_2_ (x = 0.33) thin film. Thermally induced phase transformation from reactively cosputtered CaO–CoO film leads to the formation of the final phase of Ca_x_CoO_2_. The phase transformation consists of the following steps:

CoO + O_2_ ⇒ Co_3_O_4_ (4)

Co_3_O_4_ + CaO ⇒ Ca_x_CoO_2_ (5)

The composition of Ca_x_CoO_2_ is Ca_0.33_CoO_2_ phase irrespective of the Ca:Co ratio in the as-deposited film, i.e., the Ca_0.33_CoO_2_ is the most favorable phase in this route of thermally induced solid state phase transformation. The final film of Ca_0.33_CoO_2_ is not phase pure, due to the partial presence of Co_3_O_4_ phase in the film. The Co_3_O_4_ content in the postannealed films decreases with the increase in Ca-content in the as-deposited films. The partial presence of Co_3_O_4_ phase in the Ca-rich films Ca:Co = 0.35 and 0.45 is attributed to the local enrichment of CoO phase in the as-deposited films. It is expected that the phase pure Ca_0.33_CoO_2_ can be grown by ensuring a homogeneous distribution of CoO and CaO phases in the initial sputtered deposited films.

## Supplementary Materials

The following are available online at https://www.mdpi.com/2079-4991/9/3/443/s1, Figure S1: θ –2θ XRD patterns of as-deposited CaO–CoO film (**a**) Ca:Co = 0.25, (**b**) Ca:Co = 0.35, (**c**) Ca:Co = 0.45.
