# Supplementary material for: Growth of CaxCoO2 Thin Films by A Two-Stage Phase Transformation from CaO–CoO Thin Films Deposited by Rf-Magnetron Reactive Cosputtering
Source: Nanomaterials (Basel). 2019 Mar 15;9(3):443. doi: 10.3390/nano9030443 (PMC6474102; doi:10.3390/nano9030443)
Supplement: Supplementary file 1 [file nanomaterials-09-00443-s001.pdf]

Supplementary information

Figure S1 shows θ – 2 θ XRD scan for the as-deposited films Ca:Co = 0.25, Ca:Co = 0.35 and Ca:Co = 0.45. XRD scans of all the as-deposited films are similar in appearance, albeit with varying CaO:CoO peak intensity ratios. It is evident from the XRD analyses that as deposited film consists of CaO and CoO phases.

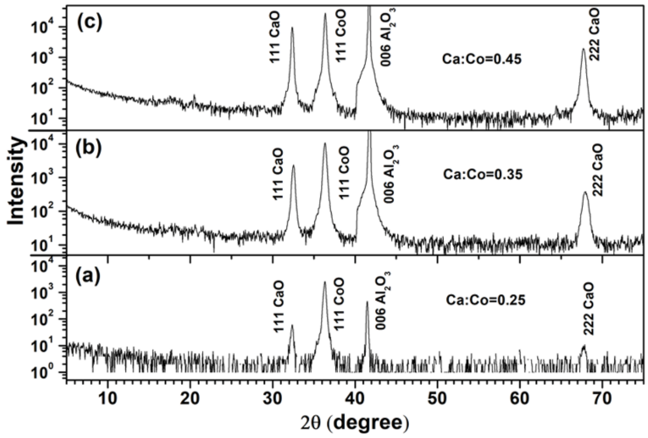

Figure S1. θ –2θ XRD patterns of as-deposited CaO–CoO film (**a**) Ca:Co = 0.25, (**b**) Ca:Co = 0.35, (**c**) Ca:Co = 0.45.
